# Tensor-Valued Diffusion MRI for Microstructural Assessment During Stereotactic Radiotherapy of Brain Metastases: A Feasibility Study

**DOI:** 10.3390/tomography12050071

**Published:** 2026-05-13

**Authors:** Minna Lerner, Patrik Brynolfsson, Filip Szczepankiewicz, Joakim Medin, Pia C. Sundgren, Lars E. Olsson, Sara Alkner

**Affiliations:** 1 Department of Translational Medicine, Medical Radiation Physics, Lund University, Skåne University Hospital Malmö, SE-205 02 Malmö, Sweden; 2 Department of Hematology, Oncology and Radiation Physics, Skåne University Hospital, Klinikgatan 5, SE-221 85 Lund, Sweden; 3 Hero Imaging AB, Norra Gimonäsvägen 39, SE-90738 Umeå, Sweden; 4 Department of Medical Radiation Physics, Clinical Sciences Lund, Lund University, Barngatan 4, Skåne University Hospital Lund, SE-221 85 Lund, Sweden; 5 Department of Diagnostic Radiology, Clinical Sciences Lund, Lund University, Skåne University Hospital Lund, SE-221 85 Lund, Sweden; 6 Lund BioImaging Centre (LBIC), Lund University, Klinikgatan 32, SE-221 84 Lund, Sweden; 7 Department of Clinical Sciences Lund, Oncology and Pathology, Lund University, Sölvegatan 19, BMC I12, SE-221 84 Lund, Sweden

**Keywords:** brain metastases, stereotactic radiotherapy, diffusion magnetic resonance imaging, diffusion tensor imaging, imaging biomarkers

## Abstract

Objectives: Early identification of treatment response in brain metastases remains clinically challenging. This study explores tensor-valued diffusion MRI (dMRI), specifically q-space trajectory imaging (QTI), as a novel source of early imaging biomarkers during stereotactic radiotherapy (SRT). Methods: Twenty-six patients with brain metastases were enrolled; thirteen met quality and completeness criteria for QTI analysis (10 responders, three non-responders). MRI was acquired at four time points: before SRT, before final SRT fraction, and at 3 and 6 months post-SRT. QTI-derived metrics included mean diffusivity (MD), fractional anisotropy (FA), microscopic FA (µFA), and isotropic (MKI) and anisotropic (MKA) diffusional variance. Parameter values within the tumour volume were compared pre- and during SRT and correlated with treatment response from standard MRI follow-up. Overall survival was assessed using Kaplan–Meier analysis. Results: Median survival was 12 months. QTI analysis was feasible with visible changes in the tumour tissue parameter maps over time. Statistically significant differences (*p* < 0.05) were found between responders and non-responders in FA before treatment. MKI in responders was significantly lower (*p* < 0.05) during SRT than before. Conclusions: This study presents a first exploration of QTI-derived parameters in a cohort of patients with brain metastases. We demonstrate feasibility and a scalable workflow, supporting further investigation in larger cohorts and in patients with larger or more stable lesions.

## Simple Summary

This pilot study investigates the feasibility of using advanced diffusion MRI to study treatment response of brain metastases undergoing stereotactic radiotherapy. At present, doctors have limited tools to determine early which tumours are responding well and which are not. Using tensor-valued diffusion MRI, which provides additional tissue information compared to standard diffusion, we mapped changes within tumours during and after treatment. We found differences between tumours that responded to therapy and those that did not. Although the study included only a small number of patients, these results are hypothesis-generating and may support future personalised treatment strategies while motivating larger clinical studies.

## 1. Introduction

Brain metastases, i.e., secondary brain tumours, are serious complications of systemic cancer. One of the most common treatment options is radiotherapy. More specifically, stereotactic radiotherapy (SRT) is recommended for patients with one to four brain metastases and a good performance status [1]. SRT delivers a high radiation dose to a small volume, while minimising radiation to surrounding, healthy tissue. The treatment has shown effectiveness in achieving good local control [2,3], and offers advantages such as short treatment duration and low neurotoxicity [4]. Yet, a subset of patients does not respond to treatment.

Follow-up of brain metastases after radiotherapy is conducted with conventional magnetic resonance imaging (MRI), assessing changes in tumour volume weeks to months after treatment completion [5]. Consequently, clinical MRI methods cannot distinguish responders from non-responders for months. This may delay actions on subsequent treatments for the individual patient that may benefit from this, as well as causing potential overtreatment of this fragile patient group. An early imaging biomarker, indicating non-responders early after or already during radiotherapy, could provide improved follow-up and enable more personalised treatments [6].

Attempts at finding new predictive imaging biomarkers have been made using various MRI techniques [7]. One promising example is diffusion MRI (dMRI), which is a relatively fast, non-invasive method. Because the motion of the water molecules is sensitive to changes in the tissue microstructure, it has the potential to improve our understanding of radiotherapy-related mechanisms during and after treatment, reflecting processes such as necrosis, oedema, and radiation-induced changes in cell density [8].

Conventional dMRI measures diffusion along multiple directions and calculates the mean diffusivity, often represented as the apparent diffusion coefficient (ADC). However, the ADC does not reflect the true diffusivity of water; rather, it represents an effective diffusion influenced by various factors, including restrictions from cell membranes and organelles, perfusion effects, and intra-voxel heterogeneity [9]. The potential of ADC as a predictive imaging biomarker for treatment response was highlighted more than twenty years ago [10]. Since then, several studies have reported ADC to correlate with treatment response. For instance, one study demonstrated an early increase in ADC in brain metastases to be associated with better clinical outcome after radiotherapy of 30 Gy in 10 fractions [11]. Despite its potential, ADC has not yet achieved clinical acceptance as an imaging biomarker for treatment response. One challenge is that the interpretation of ADC is non-trivial due to the many parallel biological processes induced by radiotherapy. Therefore, the simplicity of ADC might render it insufficient as a predictive biomarker on its own, especially in the brain where the tumour microenvironment is complex and dynamic.

Multidimensional dMRI, such as tensor-valued diffusion encoding, is a collection of advanced methods for probing the diffusion process, and thereby subtle changes in tissue microstructure [12,13,14]. Among the first methods to exploit tensor-valued diffusion encoding was q-space trajectory imaging (QTI) [15]. It can map several tissue characteristics by estimating the corresponding diffusion parameters based on measurements with high b-values and variable b-tensor shape [15,16]. These parameters provide a more comprehensive evaluation of the diffusion patterns, beyond conventional dMRI. While ADC maps quantify the magnitude of diffusion within a voxel, averaged over all directions, QTI separates isotropic and anisotropic diffusional variance and distinguishes microscopic from macroscopic anisotropy within each voxel [16]. Feasibility of QTI has been shown for evaluating microscopic anisotropy and tissue heterogeneity in a variety of brain tumours [17].

Despite examples in diagnostic applications, tensor-valued diffusion encoding in radiotherapy applications remains underexplored. One reason for this could be the challenges associated with the radiotherapy setup [18], where brain cancer patients are fixated in a mask potentially limiting the image quality. However, the role of MRI in radiotherapy in general has increased over the last decades and dedicated MRI scanners for radiotherapy enable new research possibilities [19]. In line with this development, the technical feasibility of tensor-valued diffusion in healthy volunteers was recently demonstrated in the radiotherapy setting [20]. Building on this prior work, this pilot study aimed to evaluate the feasibility of tensor-valued diffusion in patients with brain metastases, investigating QTI parameters longitudinally before, during, and after SRT.

## 2. Materials and Methods

### 2.1. Patient Cohort and Study Setup

This prospective pilot study initially aimed to include 20 patients; however, due to a substantial number of exclusions from the analysis discovered during the study, recruitment was extended to 26 patients. All patients received SRT for intracranial brain metastases, comprising 10 males and 16 females with a median age of 64 years (range 44–85 years). Primary diagnoses were lung cancer (*n* = 8), breast cancer (*n* = 7), malignant melanoma (*n* = 4), colon cancer (*n* = 3), kidney cancer (*n* = 2), testis cancer (*n* = 1) and unknown primary (*n* = 1). The study was approved by the National Ethical Review Board, Sweden (2020-01495), and written informed consent was obtained from all participants.

Inclusion criteria were adult patients (>18 years), no MRI contraindications, tumour diameter ≥10 mm, and a life expectancy >6 months. Prescribed absorbed dose was 30, 24, or 21 Gy in 3 fractions, depending on tumour location and size, according to clinical treatment protocols. Each patient was scheduled for four MRI examinations: before SRT, during SRT (adjacent to the final treatment fraction), and follow-up at 3 and 6 months post-SRT. In cases of personal or health-related reasons, follow-up examinations were allowed to be performed at the patient’s local hospital. These examinations did then not include QTI, and the follow-up results from the radiological report were included.

### 2.2. MRI Acquisition

MRI data was acquired on a wide bore 3T-scanner (Discovery 750w, software release DV26.0_R03 or MR30.0_R01, General Electric (GE) Healthcare, Milwaukee, WI, USA) with maximum gradient strength 44 mT/m and slew rate 200 T/m/s. All patients were scanned in treatment position, with a coil configuration of 6-channel flex coils and an 8-channel posterior array. The patients were fixated using a closed, three-point fixation mask during the first MRI. All four examinations included anatomical T2-weighted (T2w) and T1-weighted (T1w) images, both with and without gadolinium (Gd) contrast (Clariscan, GE Healthcare, 0.5 mmol/mL, dosage 0.2 mL/kg of body weight). Additionally, functional imaging was acquired using pre-contrast conventional and tensor-valued diffusion techniques. Tensor-valued diffusion encoding was acquired using a custom echo-planar imaging (EPI) sequence (sequence prototype supplied by the vendor) with acquisition matrix 80 × 80 in 27 contiguous slices, echo time (TE) 119.5 ms, repetition time (TR) 7200 ms, in-plane acceleration factor 2, and scan time 9:49 min. The strength of the diffusion encoding was b = 100, 700, 1400, 2000 s/mm^2^ acquired in 6, 6, 10, 21 directions for linear b-tensor encoding and 6, 6, 10, 15 rotations for spherical b-tensor encoding. The optimisation of the gradient waveforms has been described previously [21,22]. Deep learning-based image reconstruction (ARDL) became available after an upgrade of the MRI scanner and was utilised for the last 8 patients.

### 2.3. Image Processing

All image processing was conducted in Hero v 2024.2.0 (Hero Imaging AB, Umeå, Sweden). Tensor-valued diffusion image processing was performed after conventional image reconstruction, including denoising using MPPCA [23] and Gibbs ringing reduction using sub-voxel shifts [24]. Eddy current correction and motion correction was performed using Elastix [25] with extrapolated target volumes [26] or during ARDL reconstruction. Diffusion parameter maps were calculated using QTI with positivity constraints (QTI+) [27] implemented in DIPY v. 1.7 [28]. Five parameters were analysed in this study: mean diffusivity (MD), fractional anisotropy (FA), microscopic fractional anisotropy (µFA), and the isotropic (MKI) and anisotropic (MKA) components of the mean diffusional kurtosis. MD, analogous to ADC derived from conventional dMRI, quantifies the overall magnitude of diffusion within a voxel. FA quantifies the degree of voxel-wise diffusion anisotropy and is influenced by both intrinsic microstructural anisotropy and fibre orientation dispersion. Extending FA, µFA measures diffusion anisotropy at the microscopic level, independent of orientation dispersion. Diffusional kurtosis reflects the variance of diffusivity and captures microstructural heterogeneity arising from isotropic (MKI) and anisotropic (MKA) tissue features [16].

### 2.4. Radiotherapy

Radiotherapy treatment planning was performed by experienced dosimetrists according to local clinical routine using 2–4 arcs, single isocentre, volumetric modulated arc therapy with 6 MV photons flattening filter free in Eclipse TPS (v 15.6.5 and v 18.0.1, Varian Medical Systems, Palo Alto, CA, USA). All patients received SRT on a TrueBeam linear accelerator (v 2.7 and v 3.0, Varian Medical Systems, Palo Alto, CA, USA). Patient setup was verified using daily cone-beam CT images. For the last 8 patients, additional pre- and intra-fractional 2D X-ray images using ExacTrac Dynamic (Brainlab AG, Munich, Germany) were obtained.

### 2.5. Treatment Outcome

Local response of individual brain metastases was assessed volumetrically on the T1w + Gd images. In line with a previous study in the same field [29], the local response was categorised as a responder (including partial and complete response) if the tumour volume had decreased at least 30% compared to baseline, and as a non-responder (including stable and progressive disease) if the volume was larger, equal, or had decreased by less than 30%. The reason to use this categorization instead of the established RANO criteria for brain metastases was that the RANO classification includes assessment of new lesions, as well as clinical status and steroid use. Hence, this classification was not applicable for our study, where the primary endpoint was treatment response in the specific treated lesion. Survival was visualised using the Kaplan–Meier method, plotted in R [30].

### 2.6. Analysis

Quantitative QTI analysis was restricted to the pre- and during SRT scans due to limited availability of the sequence at follow-up examinations or minimal residual tumour tissue at later time points. Tumour volumes were delineated in the clinical workflow on contrast enhanced T1-weighted images by experienced radiation oncologists in accordance with clinical guidelines for gross tumour volume (GTV) definition. For images acquired during SRT, GTVs were transferred from the baseline MRI and subsequently adjusted when necessary, by M.L., in consultation with a radiation oncologist.

The volumes were prepared for analysis with necrotic regions excluded, using a threshold of MD > 2 µm^2^/ms. Additionally, voxels with failed parameter estimation were excluded from the analysis by incorporating thresholds according to FA, µFA > 1, and MKI, MKA > 4. Median QTI parameter values were obtained for the remaining voxels within each GTV, and basic descriptive statistics were calculated and compared.

Voxel-wise QTI parameter distributions were compared between responders and non-responders at each time point, and between pre- and during SRT within each response group using patient-level permutation tests (10,000 permutations) to account for spatial dependence among voxels within each patient. For each comparison, voxel values from all patients within a group were combined to compute a two-sample Kolmogorov–Smirnov (KS) statistic. During permutations, entire patients (and all associated voxels) were randomly reassigned to groups, and the KS statistic was recomputed. The permutation *p*-value was defined as the proportion of KS statistics obtained under permutation that were equal to or greater than the observed KS statistic, thereby estimating the probability of observing a statistic at least as extreme under the null hypothesis.

For longitudinal analyses (pre-SRT versus during-SRT), paired patient-level permutations were used. The pre/during labels were randomly retained or swapped for each patient in every permutation, after which the KS statistic was recalculated. This approach preserves patients as the independent units while making full use of the underlying voxel distributions. Statistical significance was assessed using permutation *p*-values with a significance threshold of α = 0.05.

To ensure sufficient image quality, we excluded images from the QTI analysis if they had a signal-to-noise ratio (SNR) of less than 3 in more than 80% of the voxels within the GTV at the highest b-value [20].

## 3. Results

**Figure 1:**
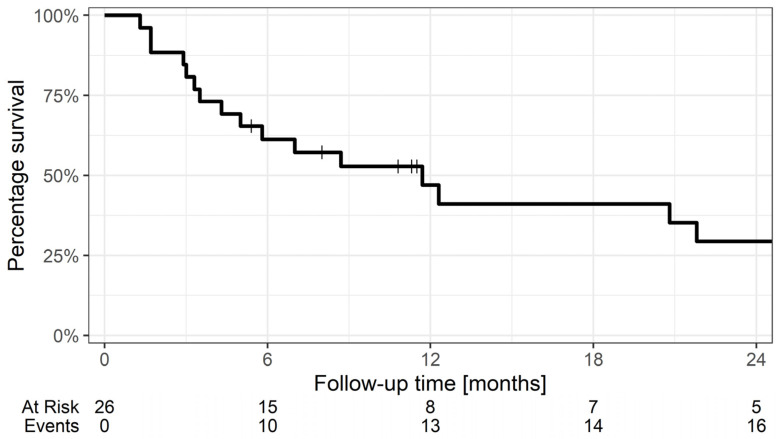
Survival plot of patients receiving SRT for brain metastases (*n* = 26). The median survival time was 12 months. Censored data points are indicated by vertical ticks. The table below the plot indicates the number of patients at risk and cumulative events at each time point.

**Figure 2:**
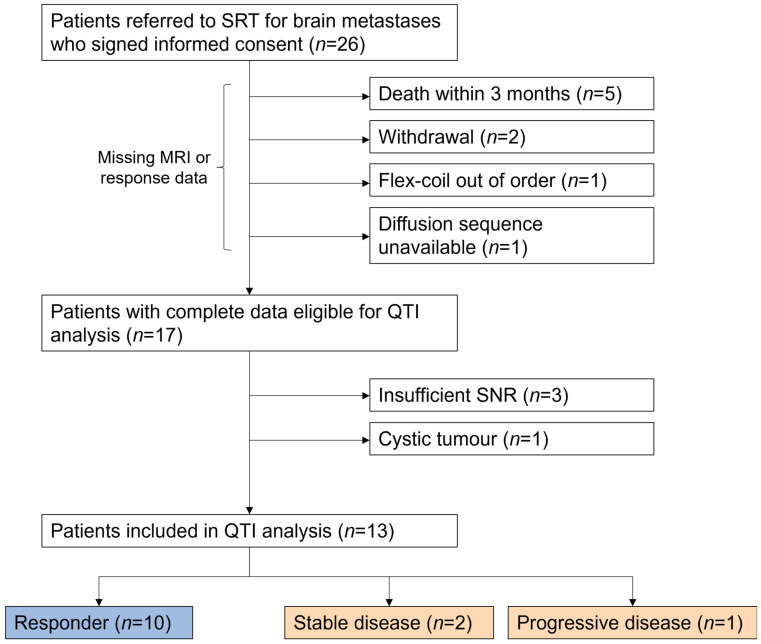
A flow diagram of patient selection for QTI analysis.

Median overall survival (*n* = 26) was 12 months (Figure 1). Prior to further analysis, nine patients were excluded due to missing MRI examinations caused by technical issues (e.g., receiver coil malfunction or temporary unavailability of study sequence following a system upgrade), withdrawal, or death. An additional four patients were excluded because of inadequate signal-to-noise ratio in the MR images (*n* = 3) or the presence of a cystic tumour (*n* = 1). After the subsequent implementation of AI-based image reconstruction, all remaining examinations met the required image quality criteria and no further exclusions were necessary. In total, QTI analysis was successfully conducted on thirteen patients, comprising six males and seven females. Patient characteristics are summarised in Table S1. A flow diagram visualises the exclusion reasons and final cohort (Figure 2).

Primary diagnoses in the analysed cohort were cancer in the breast (*n* = 4), lung (*n* = 4), colon (*n* = 2), testis (*n* = 1), malignant melanoma (*n* = 1), and unknown primary (*n* = 1). Median volume (range) of the analysed metastases, prior to thresholding irrelevant parameter values, was 7.7 cm^3^ (1.0–36.6 cm^3^) for responders and 2.3 cm^3^ (1.8–3.5 cm^3^) for non-responders.

**Figure 3:**
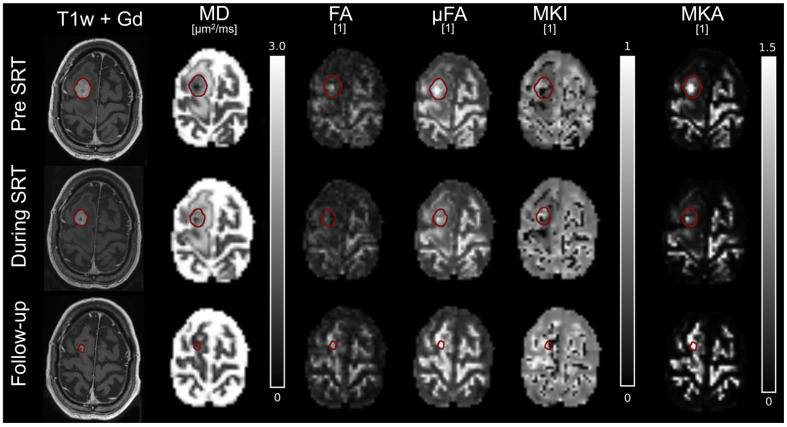
Parameter maps for a patient with primary lung cancer (responder). Parameter maps were collected pre-SRT (**top row**), during SRT after 20 Gy (**middle row**), and at 3 months follow-up (**bottom row**). The red outline indicates the contrast enhanced tumour tissue, delineated on T1w + Gd at each time point.

**Figure 4:**
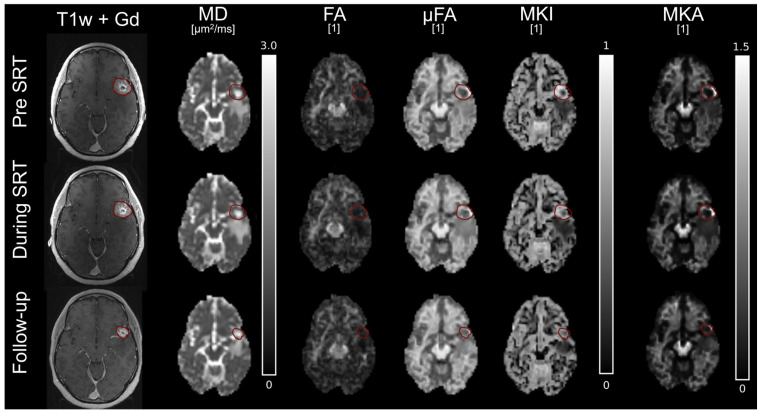
Parameter maps for a patient with primary breast cancer (responder). Parameter maps were collected pre-SRT (**top row**), during SRT after 20 Gy (**middle row**), and at 3 months follow-up (**bottom row**). The red outline indicates the contrast enhanced tumour tissue, delineated on T1w + Gd at each time point.

All maps were consistent across time on the contralateral, healthy side of the brain, as demonstrated for two responder cases in Figure 3 and Figure 4. In the first example (Figure 3), the tumour demonstrated a reduction in contrast-enhanced tumour volume between the baseline MRI pre-SRT and the MRI during SRT, with an increase in MD over time. The parameters relating to diffusion anisotropy and directional dependence (FA, µFA, and MKA) were elevated prior to SRT but decreased in intensity during SRT. Three months post-SRT, the tumour had decreased significantly in volume.

In another example (Figure 4), tissue heterogeneity (MKI) was elevated before SRT, but gradually decreased during and after SRT. The parameters related to anisotropy, µFA and MKA, were also elevated in parts of the tumour edges, which also decreased with time during and after SRT. The centre of the tumour was partly necrotic, as indicated by the non-enhanced region in the T1w + Gd images and correspondingly high MD.

**Figure 5:**
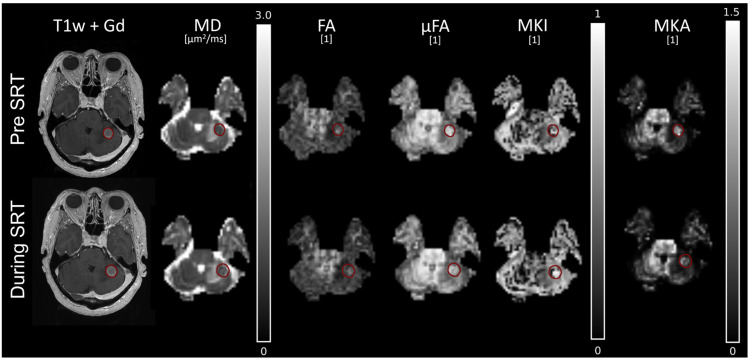
Parameter maps for a patient with primary colon cancer (non-responder). Parameter maps were collected pre-SRT (**top row**) and during SRT after 20 Gy (**bottom row**). The red outline indicates the contrast enhanced tumour tissue, delineated on T1w + Gd at each time point.

The parameter maps of a non-responder are shown in Figure 5, with an increase in tumour volume during SRT. For this patient, the anisotropy-related parameters (µFA and MKA) were again elevated prior to SRT but decreased in intensity during SRT.

Three months after SRT, standard MRI follow-up of the analysis cohort identified ten patients with tumour volume reductions of at least 30%, defined as responders. Two patients had stable diseases, and one progressive disease. For the analysis, these three were grouped together and defined as non-responders. None of the estimated QTI parameters showed statistical significance when comparing median values for responders and non-responders, or when comparing parameter values within each response group before and during SRT (Figure S1).

**Figure 6:**
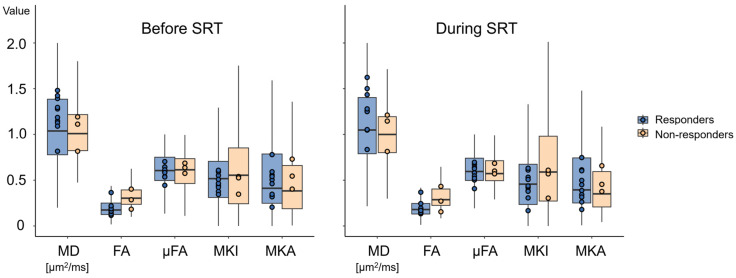
QTI parameters from the pooled voxel analysis for responders and non-responders, pre- and during SRT. Included parameters are mean diffusivity (MD), fractional anisotropy (FA), microscopic FA (µFA), and diffusional variance caused by isotropic (MKI) and anisotropic diffusion (MKA). The boxplot displays the median of the pooled voxel values by the horizontal bold line. The box indicates the 25th and 75th percentiles. Circles represent the median parameter value for each individual patient.

Voxel-wise statistical analysis was carried out at the group-level, using patient-clustered GTV voxels in the permutation tests (Figure 6). Comparing the parameter distributions (median [1st and 3rd quartiles]) before SRT demonstrated statistical significance (*p* = 0.03) for FA (0.175 [0.125, 0.251] vs. 0.303 [0.237, 0.393]) for responders and non-responders, respectively. Significance did not remain during SRT with FA (0.180 [0.131, 0.246] vs. 0.292 [0.226, 0.413]) (*p* = 0.07). In responders, MKI was significantly lower during SRT than before (*p* = 0.02). All other QTI parameter differences were non-significant (*p* > 0.05). All values are presented in Table S2.

## 4. Discussion

Developing predictive imaging biomarkers for brain metastases is a key challenge in advancing personalised radiotherapy. This pilot study explores the potential of tensor-valued dMRI parameters to address this need, providing initial, preliminary insights into microstructural changes during SRT. We were able to demonstrate feasibility of acquiring and analysing voxel-wise QTI parameters during radiotherapy in patients with brain metastases. The potentially low statistical power and the patterns from individual parameter maps across all QTI metrics suggest that the method could be capable of detecting biologically relevant changes and warrants further investigation. By using patient-level permutation testing, the present analysis respects the intrinsic spatial structure of the data and provides a statistically robust framework for future studies.

Even if sensitivity and specificity were limited in this small cohort, we observed promising trends in individual patients, supporting the potential of QTI parameters as imaging biomarkers predicting response to SRT in brain metastases. For instance, in some cases, changes in QTI parameters during SRT seemed to correspond to a simultaneous or later reduction in tumour volume. This included an increase in MD and reduced anisotropy-related parameters and suggests the presence of biologically meaningful changes that should be explored further in a larger, more powered cohort.

Higher MD could be interpreted as less dense tissue, which has been associated with less aggressive brain tumours [31,32]. Prior studies have reported associations between higher baseline or post-treatment ADC and better clinical outcome [33,34], including increased ADC during radiotherapy in responders [11]. Conversely, some studies report lower ADC in responders [35], highlighting the complexity of this metric and the need for more specific or complementary imaging parameters. Although the MD distribution of responders reached higher values in our study, the cohort was not large enough to show a statistically significant difference between responders and non-responders.

Fractional anisotropy (FA), which relates to orientation coherence of the tissue microstructure, was found to be higher in the non-responding tumours. The result should be interpreted as preliminary, given the low number of patients in each group and the difference in tumour volumes between the groups. The higher FA observed in the smaller non-responding tumours may be influenced by partial volume effects, where the surrounding white matter (characterised by intrinsically higher FA) contributes disproportionately to the signal. In smaller lesions, a larger fraction of voxels may include adjacent white matter tissue, potentially leading to an artificially elevated FA. The biological interpretation of higher FA in non-responding tissue is less intuitive, but similar results of higher FA in more aggressive primary brain tumours have been observed in previous research [31]. However, as that study investigated infiltrative gliomas rather than brain metastases, the applicability of their findings to metastatic disease should be interpreted with caution.

It is worth noting the discrepancy between the analysis of median values and the pooled analysis. When comparing metastasis-level median values, no QTI parameter reached statistical significance, while the pooled voxel analysis yielded significance for FA before SRT (*p* = 0.03) and for MKI within responders (*p* = 0.02). The median analysis inherently has limited power with only 13 patients, and particularly with only three non-responders. The voxel-wise analysis gains sensitivity by using the full parameter distributions within the GTV, but thereby also risks inflating statistical significance. Large tumours exert greater influence on the group-level distribution. It cannot be excluded that the observed FA difference is partly driven by the volume imbalance rather than by a true biological effect. Whether the FA difference would persist after adjusting for tumour volume, for instance, through volume-weighted analyses, remains an open question for future studies.

All metastases exhibited low to intermediate values of MKA, in line with a previous characterisation of different brain tumours [17]. Furthermore, the metastases exhibited intermediate microscopic heterogeneity, as measured by MKI. All parameter values are within expected ranges, both compared to white matter and to previously reported values in tumours. More detailed interpretation of the QTI parameters would require association with structural characteristics from histology, which is outside the scope of this study. Future studies should also consider a formal interobserver study to assess variability in the derived parameters.

The QTI method requires sufficient SNR for reliable parameter estimation, which can be challenging in small metastases or in regions affected by susceptibility artefacts. To ensure data reliability, a strict exclusion criterion was applied (requiring SNR > 3 for 80% of the GTV). The 3 mm isotropic voxel resolution was a compromise to achieve sufficient SNR while adhering to practical constraints. An upgrade of the MRI scanner during the study improved data quality with deep learning-based reconstruction and noise reduction (ARDL), reducing exclusions due to poor SNR. Of the 13 patients, eight were scanned with ARDL. While this upgrade was essential for achieving sufficient image quality, it also represents a mid-protocol change that warrants consideration. ARDL alters the noise characteristics of the reconstructed images and may influence quantitative diffusion parameters in ways that have not been fully characterised for tensor-valued diffusion encoding. Preliminary results indicate that the diffusion parameters remain stable despite denoising [36], but systematic differences between scans reconstructed with and without ARDL cannot be excluded. Future studies should investigate QTI parameters quantitatively across different reconstruction pipelines. More broadly, improvements such as high-performance coils and dMRI-specific noise reduction techniques [37] could further enhance imaging quality.

The clinical characteristics of the cohort imposed some limitations. Despite selecting patients with an expected survival of at least 6 months, five patients did not survive the 3-month follow-up, and five more died before 6 months. Such variation in survival aligns with previous studies [38], but the high mortality rate reduced the final sample size and statistical power. The heterogeneity of primary diagnosis in this cohort represents an additional potential confounder. The current study was not powered to assess differences in parameter values or treatment response by primary diagnosis due to the limited sample size.

Another challenge was the timing of imaging. Mahmood et al. [29] reported that ADC changes became detectable after approximately 21 Gy delivered in 7 fractions of whole-brain radiotherapy, corresponding to a modest biologically effective dose. In contrast, the during SRT scan in the present study was acquired after 20 Gy in 2 fractions, corresponding to a substantially higher biologically effective dose. This difference in fractionation limits direct comparison between the two studies. There is no consensus on the optimal timing for response prediction [7], and the choice of scanning during treatment alongside follow-up at 3 and 6 months was guided by practical considerations and patient convenience.

Several specific statistical limitations should be acknowledged. With only three non-responders among 13 patients, the number of unique group assignments in the between-group permutation test is limited to C(13,3) = 286. Furthermore, no correction for multiple comparisons was applied across the five QTI parameters tested. Together with the volume difference between groups, the heterogeneity of primary diagnosis, and the potential for ARDL-related batch effects discussed above, these factors reinforce that the present results should be interpreted as preliminary yet hypothesis-generating. The absence of statistically significant differences should not be taken as evidence of absence and does not preclude clinically meaningful effects that a larger, homogenous cohort might detect.

## 5. Conclusions

The findings of this pilot study indicate that implementing tensor-valued dMRI for patients with brain metastases is challenging yet feasible. Improved understanding of tumour sensitivity to SRT, derived from dMRI and QTI-based metrics, require further investigation but could potentially help individualise treatments in the future. Despite limitations, this study provides important initial feasibility data and establishes a workflow that can be applied and refined in larger cohorts.

## Data Availability

The data supporting the findings of this study are not publicly available due to restrictions imposed by the ethical approval and participant confidentiality. Data may be available from the corresponding author upon reasonable request and with appropriate ethical clearance.

## Supplementary Materials

The following supporting information can be downloaded at: https://www.mdpi.com/article/10.3390/tomography12050071/s1. Figure S1: QTI parameter median values; Table S1: Patient characteristics, Table S2: Parameter values from QTI pooled voxel analysis.

## Abbreviations

The following abbreviations are used in this manuscript:

ADC	Apparent diffusion coefficient
ARDL	AIR recon DL
dMRI	Diffusion magnetic resonance imaging
FA	Fractional anisotropy
GTV	Gross tumour volume
KS	Kolmogorov–Smirnov
MD	Mean diffusivity
MKA	Anisotropic mean diffusional kurtosis
MKI	Isotropic mean diffusional kurtosis
MRI	Magnetic resonance imaging
QTI	Q-space trajectory imaging
SNR	Signal-to-noise ratio
SRT	Stereotactic radiotherapy
TE	Echo time
TR	Repetition time
µFA	Microscopic fractional anisotropy

## References

[1] Gondi V., Bauman G., Bradfield L., Burri S.H., Cabrera A.R., Cunningham D.A., Eaton B.R., Hattangadi-Gluth J.A., Kim M.M., Kotecha R. (2022). Radiation Therapy for Brain Metastases: An ASTRO Clinical Practice Guideline. Pract. Radiat. Oncol..

[2] Soliman H., Das S., Larson D.A., Sahgal A. (2016). Stereotactic radiosurgery (SRS) in the modern management of patients with brain metastases. Oncotarget.

[3] Redmond K.J., Gui C., Benedict S., Milano M.T., Grimm J., Vargo J.A., Soltys S.G., Yorke E., Jackson A., El Naqa I. (2021). Tumor Control Probability of Radiosurgery and Fractionated Stereotactic Radiosurgery for Brain Metastases. Int. J. Radiat. Oncol. Biol. Phys..

[4] Brown P.D., Jaeckle K., Ballman K.V., Farace E., Cerhan J.H., Anderson S.K., Carrero X.W., Barker F.G., Deming R., Burri S.H. (2016). Effect of Radiosurgery Alone vs Radiosurgery with Whole Brain Radiation Therapy on Cognitive Function in Patients with 1 to 3 Brain Metastases: A Randomized Clinical Trial. JAMA.

[5] Lin N.U., Lee E.Q., Aoyama H., Barani I.J., Barboriak D.P., Baumert B.G., Bendszus M., Brown P.D., Camidge D.R., Chang S.M. (2015). Response assessment criteria for brain metastases: Proposal from the RANO group. Lancet Oncol..

[6] Mehrabian H., Detsky J., Soliman H., Sahgal A., Stanisz G.J. (2019). Advanced Magnetic Resonance Imaging Techniques in Management of Brain Metastases. Front. Oncol..

[7] Hu J., Xie X., Zhou W., Hu X., Sun X. (2023). The emerging potential of quantitative MRI biomarkers for the early prediction of brain metastasis response after stereotactic radiosurgery: A scoping review. Quant. Imaging Med. Surg..

[8] Shah A.D., Shridhar Konar A., Paudyal R., Oh J.H., LoCastro E., Nunez D.A., Swinburne N., Vachha B., Ulaner G.A., Young R.J. (2021). Diffusion and Perfusion MRI Predicts Response Preceding and Shortly After Radiosurgery to Brain Metastases: A Pilot Study. J. Neuroimaging.

[9] Le Bihan D. (2013). Apparent diffusion coefficient and beyond: What diffusion MR imaging can tell us about tissue structure. Radiology.

[10] Mardor Y., Pfeffer R., Spiegelmann R., Roth Y., Maier S.E., Nissim O., Berger R., Glicksman A., Baram J., Orenstein A. (2003). Early detection of response to radiation therapy in patients with brain malignancies using conventional and high b-value diffusion-weighted magnetic resonance imaging. J. Clin. Oncol..

[11] Mahmood F., Hjorth Johannesen H., Geertsen P., Hansen R.H. (2020). Diffusion MRI outlined viable tumour volume beats GTV in intra-treatment stratification of outcome. Radiother. Oncol..

[12] Uddin M.N., Singh M.V., Faiyaz A., Szczepankiewicz F., Nilsson M., Boodoo Z.D., Sutton K.R., Tivarus M.E., Zhong J., Wang L. (2024). Tensor-valued diffusion MRI detects brain microstructural abnormalities in HIV infected individuals with cognitive impairment. Sci. Rep..

[13] Cho E., Baek H.J., Szczepankiewicz F., An H.J., Jung E.J. (2024). Imaging evaluation focused on microstructural tissue changes using tensor-valued diffusion encoding in breast cancers after neoadjuvant chemotherapy: Is it a promising way forward?. Gland. Surg..

[14] Teh I., Shelley D., Boyle J.H., Zhou F., Poenar A.M., Sharrack N., Foster R.J., Yuldasheva N.Y., Parker G.J.M., Dall’Armellina E. (2023). Cardiac q-space trajectory imaging by motion-compensated tensor-valued diffusion encoding in human heart in vivo. Magn. Reson. Med..

[15] Westin C.F., Knutsson H., Pasternak O., Szczepankiewicz F., Ozarslan E., van Westen D., Mattisson C., Bogren M., O’Donnell L.J., Kubicki M. (2016). Q-space trajectory imaging for multidimensional diffusion MRI of the human brain. Neuroimage.

[16] Szczepankiewicz F., van Westen D., Englund E., Westin C.F., Stahlberg F., Latt J., Sundgren P.C., Nilsson M. (2016). The link between diffusion MRI and tumor heterogeneity: Mapping cell eccentricity and density by diffusional variance decomposition (DIVIDE). Neuroimage.

[17] Nilsson M., Szczepankiewicz F., Brabec J., Taylor M., Westin C.F., Golby A., van Westen D., Sundgren P.C. (2020). Tensor-valued diffusion MRI in under 3 minutes: An initial survey of microscopic anisotropy and tissue heterogeneity in intracranial tumors. Magn. Reson. Med..

[18] Gurney-Champion O.J., Mahmood F., van Schie M., Julian R., George B., Philippens M.E.P., van der Heide U.A., Thorwarth D., Redalen K.R. (2020). Quantitative imaging for radiotherapy purposes. Radiother. Oncol..

[19] Goodburn R.J., Philippens M.E.P., Lefebvre T.L., Khalifa A., Bruijnen T., Freedman J.N., Waddington D.E.J., Younus E., Aliotta E., Meliado G. (2022). The future of MRI in radiation therapy: Challenges and opportunities for the MR community. Magn. Reson. Med..

[20] Brynolfsson P., Lerner M., Sundgren P.C., Jamtheim Gustafsson C., Nilsson M., Szczepankiewicz F., Olsson L.E. (2022). Tensor-valued diffusion magnetic resonance imaging in a radiotherapy setting. Phys. Imaging Radiat. Oncol..

[21] Sjölund J., Szczepankiewicz F., Nilsson M., Topgaard D., Westin C.F., Knutsson H. (2015). Constrained optimization of gradient waveforms for generalized diffusion encoding. J. Magn. Reson..

[22] Szczepankiewicz F., Westin C.F., Nilsson M. (2019). Maxwell-compensated design of asymmetric gradient waveforms for tensor-valued diffusion encoding. Magn. Reson. Med..

[23] Olesen J.L., Ianus A., Østergaard L., Shemesh N., Jespersen S.N. (2023). Tensor denoising of multidimensional MRI data. Magn. Reson. Med..

[24] Kellner E., Dhital B., Kiselev V.G., Reisert M. (2016). Gibbs-ringing artifact removal based on local subvoxel-shifts. Magn. Reson. Med..

[25] Klein S., Staring M., Murphy K., Viergever M.A., Pluim J.P. (2010). elastix: A toolbox for intensity-based medical image registration. IEEE Trans. Med. Imaging.

[26] Nilsson M., Szczepankiewicz F., van Westen D., Hansson O. (2015). Extrapolation-Based References Improve Motion and Eddy-Current Correction of High B-Value DWI Data: Application in Parkinson’s Disease Dementia. PLoS ONE.

[27] Herberthson M., Boito D., Haije T.D., Feragen A., Westin C.F., Özarslan E. (2021). Q-space trajectory imaging with positivity constraints (QTI+). Neuroimage.

[28] Garyfallidis E., Brett M., Amirbekian B., Rokem A., van der Walt S., Descoteaux M., Nimmo-Smith I. (2014). Dipy, a library for the analysis of diffusion MRI data. Front. Neuroinform..

[29] Mahmood F., Johannesen H.H., Geertsen P., Hansen R.H. (2017). Repeated diffusion MRI reveals earliest time point for stratification of radiotherapy response in brain metastases. Phys. Med. Biol..

[30] R Core Team (2023). R: A Language and Environment for Statistical Computing.

[31] Miloushev V.Z., Chow D.S., Filippi C.G. (2015). Meta-Analysis of Diffusion Metrics for the Prediction of Tumor Grade in Gliomas. Am. J. Neuroradiol..

[32] Hayashida Y., Hirai T., Morishita S., Kitajima M., Murakami R., Korogi Y., Makino K., Nakamura H., Ikushima I., Yamura M. (2006). Diffusion-weighted imaging of metastatic brain tumors: Comparison with histologic type and tumor cellularity. AJNR Am. J. Neuroradiol..

[33] Zhao L., Zhao M., Liu J., Yang H., Zhou X., Wen C., Li G., Duan Y. (2021). Mean apparent diffusion coefficient in a single slice may predict tumor response to whole-brain radiation therapy in non-small-cell lung cancer patients with brain metastases. Eur. Radiol..

[34] Chen Z., Zu J., Li L., Lu X., Ni J., Xu J. (2017). Assessment of stereotactic radiosurgery treatment response for brain metastases using MRI based diffusion index. Eur. J. Radiol. Open.

[35] Jakubovic R., Zhou S., Heyn C., Soliman H., Zhang L., Aviv R., Sahgal A. (2016). The predictive capacity of apparent diffusion coefficient (ADC) in response assessment of brain metastases following radiation. Clin. Exp. Metastasis.

[36] Brynolfsson P., Rashid I., Lerner M., Olsson L.E. PG085 Denoising for High-Resolution Tensor-Valued Diffusion on Medium Gradient MRI Scanners. Proceedings of the European Society for Magnetic Resonance in Medicine and Biology (ESMRMB).

[37] Manzano Patron J.P., Moeller S., Andersson J.L.R., Ugurbil K., Yacoub E., Sotiropoulos S.N. (2024). Denoising diffusion MRI: Considerations and implications for analysis. Imaging Neurosci..

[38] Brown P.D., Ballman K.V., Cerhan J.H., Anderson S.K., Carrero X.W., Whitton A.C., Greenspoon J., Parney I.F., Laack N.N.I., Ashman J.B. (2017). Postoperative stereotactic radiosurgery compared with whole brain radiotherapy for resected metastatic brain disease (NCCTG N107C/CEC·3): A multicentre, randomised, controlled, phase 3 trial. Lancet Oncol..

