# Supplementary material for: Tensor-Valued Diffusion MRI for Microstructural Assessment During Stereotactic Radiotherapy of Brain Metastases: A Feasibility Study
Source: Tomography. 2026 May 13;12(5):71. doi: 10.3390/tomography12050071 (PMC13211140; doi:10.3390/tomography12050071)
Supplement: Supplementary file 1 [file tomography-12-00071-s001.zip › tomography-4185391-supplementary.pdf]

Tomography

Supplementary material: Tensor-Valued Diffusion MRI for Microstructural Assessment During Stereotactic Radiotherapy of Brain Metastases: A Feasibility Study

Minna Lerner [1,2,*], Patrik Brynolfsson [1,2,3], Filip Szczepankiewicz [4], Joakim Medin [2,4], Pia C. Sundgren [5,6], Lars E. Olsson [1,2] and Sara Alkner [2,7]

[1] Department of Translational Medicine, Medical Radiation Physics, Lund University, Skåne University Hospital Malmö, SE-205 02 Malmö, Sweden;
[2] Department of Hematology, Oncology and Radiation Physics, Skåne University Hospital, Klinikgatan 5, SE-221 85 Lund, Sweden;
[3] Hero Imaging AB, Norra Gimonäsvägen 39, SE-90738 Umeå, Sweden;
[4] Department of Medical Radiation Physics, Clinical Sciences Lund, Lund University, Barngatan 4, Skåne University Hospital Lund, SE-221 85 Lund, Sweden;
[5] Department of Diagnostic Radiology, Clinical Sciences Lund, Lund University, Skåne university hospital Lund, SE-221 85 Lund, Sweden;
[6] Lund BioImaging Centre (LBIC), Lund University, Klinikgatan 32, SE-221 84 Lund, Sweden;
[7] Department of Clinical Sciences Lund, Oncology and Pathology, Lund University, Sölvegatan 19, BMC I12, SE-221 84 Lund, Sweden
* Correspondence: minna.lerner@med.lu.se

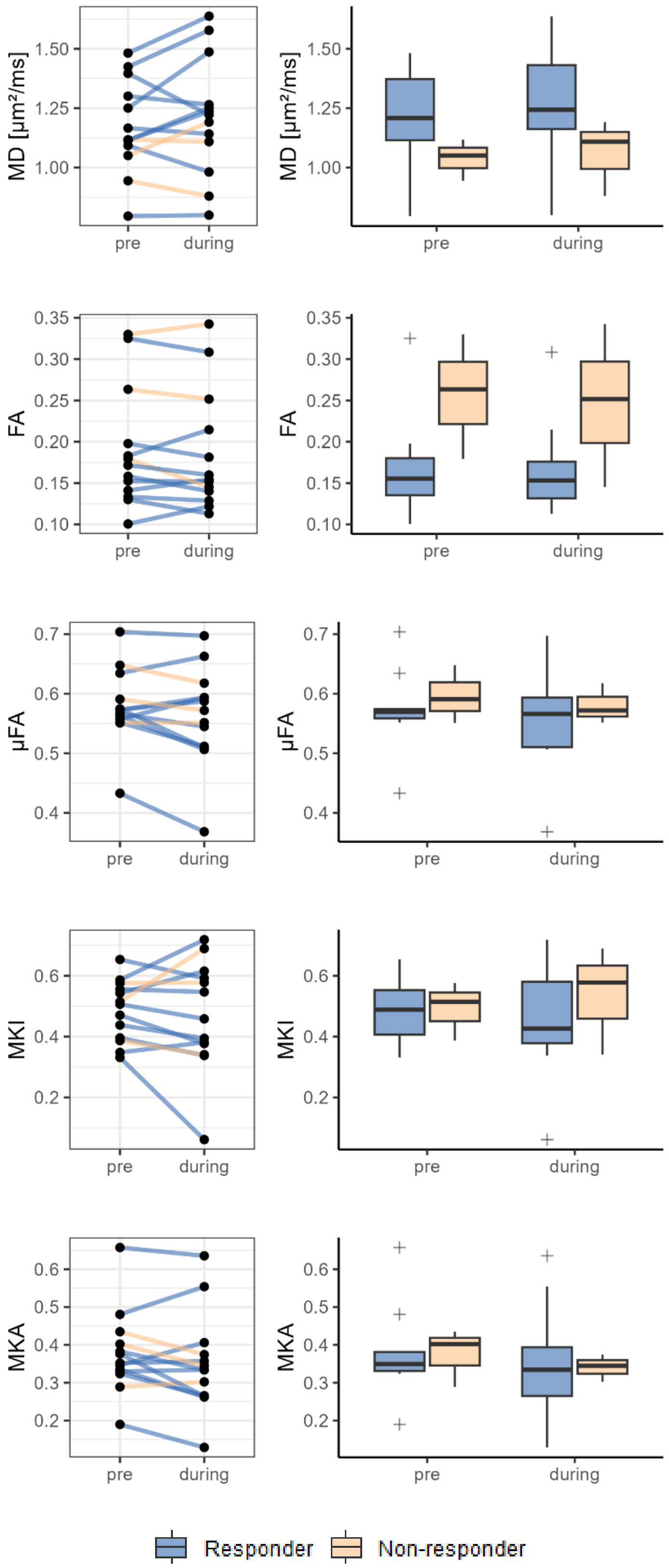

Figure S1. QTI parameter median values presented for each patient in the left column, indicating changes between MRI examinations pre- and during SRT (individual lines per patient). Each row corresponds to a different parameter; mean diffusivity (MD), fractional anisotropy (FA), microscopic FA (µFA) and diffusional variance caused by isotropic (MKI) and anisotropic diffusion (MKA). The second column shows the results from pre-SRT and during-SRT results. The horizontal bold line within each box represents the median of the population. Outliers are represented by a plus symbol.

Table S1. Patient summary from the QTI analysis. [F = female, M = male, ARDL = AI-based image reconstruction]

ID	Gender	Age (yr)	Primary	Prescribed dose	Recon	Outcome
1	F	60	Breast cancer	30 Gy / 3 fx	Conventional	Responder
4	F	61	Lung cancer	30 Gy / 3 fx	Conventional	Responder
11	F	47	Breast cancer	30 Gy / 3 fx	Conventional	Responder
16	M	63	Melanoma	30 Gy / 3 fx	Conventional	Responder
18	M	53	Lung cancer	30 Gy / 3 fx	Conventional	Responder
19	F	56	Unknown	21 Gy / 3 fx	ARDL	Responder
20	M	58	Lung cancer	30 Gy / 3 fx	ARDL	Responder
21	M	80	Lung cancer	30 Gy / 3 fx	ARDL	Responder
22	F	53	Breast cancer	24 Gy / 3 fx	ARDL	Non-responder (stable)
23	F	64	Colon cancer	30 Gy / 3 fx	ARDL	Non-responder (stable)
24	M	55	Testis cancer	30 Gy / 3 fx	ARDL	Responder
25	F	67	Breast cancer	21 Gy / 3 fx	ARDL	Responder
26	M	71	Colon cancer	30 Gy / 3 fx	ARDL	Non-responder

Table S2. Parameter values (median [1st and 3rd quartiles]) from QTI pooled voxel analysis for responders and non-responders, before and during SRT. Patient-level permutation tests with Kolmogorov-Smirnov statistic was performed comparing responders and non-responders before and during SRT. Responders and non-responders were also evaluated separately, comparing parameter values before and during SRT. All *p*-values are presented, with significance indicated by an asterisk (*).

	Pre SRT		***During SRT***	
	**Responder**	**Non-resp.**	**Responder**	**Non-resp.**
**MD [μm²/ms]**	1.036 [0.777, 1.385]	1.008 [0.822, 1.217]	1.047 [0.787, 1.402]	0.992 [0.793, 1.191]
**FA**	0.175 [0.125, 0.251]	0.303 [0.237, 0.393]	0.180 [0.131, 0.246]	0.292 [0.226, 0.413]
**μFA**	0.605 [0.497, 0.751]	0.613 [0.464, 0.735]	0.595 [0.497, 0.740]	0.580 [0.495, 0.725]
**MKI**	0.517 [0.311, 0.707]	0.554 [0.243, 0.852]	0.459 [0.236, 0.676]	0.661 [0.282, 1.065]
**MKA**	0.412 [0.249, 0.788]	0.383 [0.188, 0.660]	0.395 [0.251, 0.746]	0.362 [0.208, 0.614]

	Responder vs non-resp.		***Pre SRT vs during SRT***	
	**Pre SRT**	**During SRT**	**Responders**	**Non-resp.**
	p-value	*p*-value	*p*-value	*p*-value
**MD [μm²/ms]**	0.866	0.894	0.963	1
**FA**	0.031*	0.075	0.731	0.249
**μFA**	0.930	0.975	0.730	0.504
**MKI**	0.859	0.598	0.019*	0.495
**MKA**	0.966	0.950	0.657	0.504
